# Evolution and Predictors of Right Ventricular Failure in Fontan Patients: A Case-Control Study

**DOI:** 10.3390/jcm14134602

**Published:** 2025-06-29

**Authors:** Hannah S. Kim, Ginnie Abarbanell, Kathleen Simpson, Aaron M. Abarbanell, Pirooz Eghtesady, Philip T. Levy, Gautam K. Singh

**Affiliations:** 1 Department of Pediatrics, Hackensack Meridian School of Medicine, HMH Joseph M. Sanzari Children’s Hospital, Hackensack, NJ 07601, USA; hannah.kim@hmhn.org; 2 Department of Pediatrics, UT Health, San Antonio, TX 78229, USA; abarbanell@uthscsa.edu; 3 Department of Pediatrics, University of Colorado Denver, Children’s Hospital of Colorado, Denver, CO 80045, USA; kathleen.simpson@childrenscolorado.org; 4 Department of Cardiothoracic Surgery, UT Health, San Antonio, TX 78229, USA; abarbanella@uthscsa.edu; 5 Pediatric Cardiothoracic Surgery, Saint. Louis Children’s Hospital, Washington University School of Medicine, Saint Louis, MO 63110, USA; eghtesady670@wustl.edu; 6 Division of Newborn Medicine, Boston Children’s Hospital, Department of Pediatrics, Harvard Medical School, Boston, MA 02115, USA; 7 Division of Pediatric Cardiology, Children’s Hospital Michigan and Central Michigan University, Detroit, MI 48201, USA; gsingh3@dmc.org

**Keywords:** right ventricular failure, fontan failure, single ventricle, heart transplant, pediatrics

## Abstract

**Background:** Patients with single right ventricular morphology (SRV) may exhibit impaired function with increased morbidity, mortality, and need for cardiac transplant due to progressive SRV failure after the Fontan procedure. The aim of the study was to longitudinally characterize the cardiac mechanics and trajectory of disease evolution of SRV failure in Fontan patients. **Methods**: We performed a case-controlled longitudinal study of 52 patients who underwent extracardiac Fontan palliation for SRV between 1994 and 2015 and compared echocardiographic measures of right ventricular (RV) function, RV-systemic vascular coupling and ventricular remodeling between patients who required heart transplants due to SRV failure (study group, *n* = 26) and those who did not (control group, *n* = 26). To define the trajectory, measurements were obtained at four matching time points equivalent in duration from Fontan. **Results**: RV circumferential shortening function declined in both groups over the time period, but was significantly lower (*p* < 0.01) in the study group farther from the Fontan. RV-systemic vascular coupling, assessed by systolic time interval measures and RV work, was preserved in the control group, but significantly altered (*p* < 0.001) in the study group. Relative wall thickness decreased, and the minor/major-axis ratio, as an index of ventricular geometry, increased in the study group, but both remained stable in the control group. **Conclusions**: This study suggests that positive ventricular remodeling with enhanced circumferential systolic function, and preserved RV-vascular coupling, appear to be adaptive and protective mechanisms against RV failure in Fontan with SRV. These indices of cardiac mechanics may serve as clinically relevant quantifiable markers of disease evolution, and early indicators for therapeutic intervention.

## 1. Introduction

The long-term outcome for infants with single ventricle physiology has remarkably improved from a certain early death to an appreciable prospect of survival to adulthood with staged Fontan palliation [1]. However, patients with single right ventricular morphology (SRV) may still exhibit impaired systolic and diastolic function through stages of Fontan palliation, and beyond [2,3]. It is recognized that 25% of patients have a risk of late SRV failure after the Fontan procedure and the need for cardiac transplant [1,2,3]. There are several proposed causes for systemic right ventricular (RV) failure in patients with SRV, including contractility–afterload mismatch (uncoupling mechanism) from a lack of a compensatory response in RV performance to increases in afterload, but the cardiovascular mechanisms that account for the adverse outcome are not well understood [4].

In SRV physiology, the challenge for the ventricle is to remain hemodynamically coupled to the systemic circulation. Initially, the RV adapts to the increasing vascular load by augmenting contractility to maintain blood flow. Important adaptive mechanisms that enhance contractile capabilities of the RV include muscle hypertrophy with increased wall thickness. This early increase in ventricular mass is proportional to the rise in ventricular volume, known as concentric remodeling. Prolonged exposure to increased afterload and progressive pressure loading on the RV can lead to maladaptive ventricular remodeling where the hypertrophic process will be halted and stroke volume decreases. The ventricular hypertrophy will increase disproportionately to ventricular volume, referred to as eccentric remodeling [5]. To maintain cardiac output and preserve stroke volume, the RV dilates and the heart rate increases. The consequence of these adaptive responses is an increase in myocyte stress with further dilation and changes in septal wall morphology, which further impairs RV function [5].

In single ventricle physiology, the RV may adapt with Fontan patients reaching adulthood and not requiring a cardiac transplant. However, in those with SRV failure after the Fontan procedure [1,2,3], the deficient identification of the key adaptive mechanistic underpinnings of SRV failure has limited the development of clinically relevant noninvasive quantifiable markers of disease evolution in Fontan patients. We hypothesize that RV failure following Fontan palliation results from maladaptive remodeling of the RV as a systemic ventricle. In addition, we postulate that echocardiographic measures of RV performance and RV-vascular coupling may predict RV heart failure after the Fontan procedure. Accordingly, the aim of this study was to longitudinally characterize RV remodeling and function with echocardiography to discern the disease evolution of SRV in Fontan patients who subsequently required cardiac transplant.

## 2. Methods

### 2.1. Study Design

We performed a retrospective case-control longitudinal study of cardiac mechanics in two cohorts of patients who had completed staged Fontan palliation for univentricular hearts or RV morphology at St. Louis Children’s Hospital between January 2004 and August 2015.

### 2.2. Study Population

We conducted a search of our institutional database of single ventricles to identify (1) a study cohort of patients who received orthotopic heart transplant (OHT) due to SRV failure after the Fontan palliation and (2) a control cohort of Fontan patients who did not develop SRV failure matched 1:1 with a patient in the study group for age, gender, body surface area, cardiac defect, age at Fontan palliation, type of surgical palliations, and surgical era. Patients were included if they had a single ventricle due to (a) hypoplastic left heart syndrome (HLHS) with mitral and aortic atresia/stenosis, (b) double outlet right ventricle (DORV) with mitral and aortic atresia/hypoplasia and hypoplastic LV, or (c) right-dominant unbalanced atrioventricular canal (UAVC). All patients underwent extracardiac Fontan with follow-up, and had adequate images for echocardiographic measurements. Those with inadequate images precluding precise measurements were excluded from the study. Medical records were reviewed for demographic data, anatomic lesions, timing of stages of Fontan palliation, surgical details including cardiopulmonary bypass and aortic cross-clamp time, dates of listing for and completion of OHT, and serial echocardiograms with accompanying hemodynamics at studies. The institutional review board at Washington University School of Medicine approved all components of the study.

### 2.3. Echocardiographic Examination

**Figure 1:**
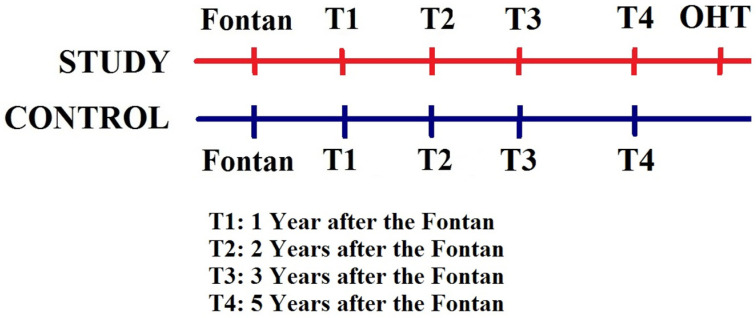
Timeline of Study. Time 1: 1 year after the Fontan procedure; Time 2: 2 years after the Fontan; Time 3: 3 years after the Fontan procedure (and around the listing date for heart transplant in the study group); Time 4: 5 years after the Fontan procedure.

Two-dimensional echocardiography with Doppler imaging was performed using a commercially available ultrasound imaging system (Philips iE33, Philips Medical Systems, Andover, MA, USA). Echocardiographic images were acquired with a standardized image acquisition protocol, stored for offline analysis (Digisonics Inc., Houston, TX, USA), and analyzed from four time points equivalent in duration from Fontan (Figure 1). Two investigators measured each of the parameters to assess for intra- and inter-observer reliability.

### 2.4. Assessment of RV Function

**Figure 2:**
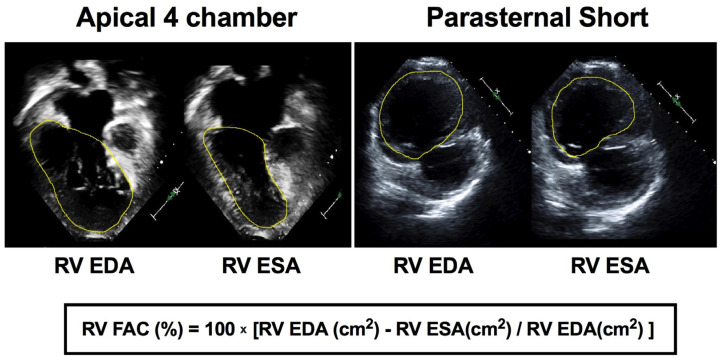
Fractional area of change (FAC). RV FAC was derived by tracing the area bound by endocardial borders of the right ventricle at end-diastole (EDA) and end-systole (ESA) in the apical four-chamber view and the parasternal short-axis view. RV FAC was calculated as 100 × EDA-ESA/EDA and expressed as a percentage.

RV longitudinal function was characterized by tricuspid annular plane systolic excursion (TAPSE, mm) and percent fractional area of change (FAC, %) [6]. TAPSE was calculated as the change in distance from the lateral annulus of the tricuspid or right AV valve to a constant fixed apical point at end-diastole to end-systole (Figure S1) [7]. Since the RV may remodel to globular geometry in SRV physiology, we conceptualized, based on circumferential fiber shortening as a component of ventricular function [8], that circumferential shortening may provide an additional index of RV function. We measured RV end-systolic area (RVESA) and RV end-diastolic area (RVEDA) in the RV-focused apical four-chamber view for longitudinal function and the parasternal short-axis view for “circumferential” function at a standardized anterior papillary muscle level located at the mid-cavity level by manual endocardial area delineation to compute FAC as 100 × [RVEDA (cm^2^) − RVESA (cm^2^)]/RVED (cm^2^)] (Figure 2) [6,9].

Two-dimensional measurement of the right atrioventricular valve (AV) annulus was measured from a four-chamber view from hinge point to hinge point at end-diastole. Right AV valve (RAVV) and its regurgitation were assessed qualitatively by all investigators. Using the size of the RAVV jet obtained at the Nyquist limit of 50–70 cm/s on color Doppler, we graded it into trivial, mild, moderate, and severe [10].

### 2.5. Assessment of RV-Vascular Coupling

RV systolic time intervals characterize the balance between intrinsic myocardial contractility, ventricular preload, and afterload [11]. In this study, we evaluated RV pre-ejection time (RV-PET) and RV ejection time (RVET). RV-PET was measured from the Q wave of the electrocardiogram tracing to the beginning of the neo-aortic Doppler waveform. RVET was measured from the beginning to the termination of the neo-aortic flow spectrum (Figure S2). The ratio of PET/RVET was calculated and compared between groups.

RV work is a comprehensive assessment of both static and dynamic afterload, and RV-vascular coupling describes a change in potential and kinetic energy between these two entities that provides insight into how RV contractility adapts to changes in afterload [5,12]. RV-vascular coupling is equal to the work expended by a force through displacement [12]. Since pressure is the force applied per unit area, we used RV systolic pressure (RVSP), rather than force, to describe the influences upon fluid behaviors (Poiseuille’s law). Due to imprecise correlation of tricuspid regurgitation-based Doppler-estimated RVSP with cardiac catheterization indices [13], we used systemic systolic blood pressure (SBP) as a surrogate for RVSP. TAPSE was substituted for displacement, and the relationship of TAPSE to RVSP was utilized as an index of RV work in the setting of RV-vascular coupling [12].

### 2.6. Index of RV Remodeling

RV remodeling can manifest as RV dilatation, hypertrophy, and/or altered chamber configuration [14] that develop in response to change in loading conditions. Since the change in RV geometry is predictive of the prognosis in cardiac lesions [15], we measured relative wall thickness (RWT) and geometric changes in the major and minor axes and chamber configuration. We acquired RWT from the RV-focused short-axis view [16] using the ratio of twice the RV inferolateral wall thickness (ILWT) constituted by the compacted myocardium, to the RV internal diameter (RVID) measured at end-diastole at the level of the anterior and septal papillary muscles (Figure S3), instead of RV end-diastolic volume [16]. We utilized the initial RWT to compare the subsequent RWT measured at the time points to discern the progression of RV remodeling in each group. Major axis was determined from the largest longitudinal length from the mid-point of the right AV valve annulus to the RV apex in the apical four-chamber view. Minor axis was the mid-RV internal dimension at the level of the anterior and septal papillary muscles in short-axis view at end-diastole as per American Society of Echocardiography guidelines (Figure S4) [17].

### 2.7. Statistical Analyses

All data is expressed as either median with interquartile ranges, percentages, and/or mean ± SD. Continuous variables were tested for normality using the Kolmogorov-Smirnov test and a histogram illustration of the data. Two group analyses for continuous variables were conducted using a student t-test or the Mann–Whitney U test as appropriate. All outcome variables with non-normal distributions were analyzed in simple comparisons using Wilcoxon rank-sum tests or Kruskal–Wallis one-way analysis of variance for tests with more than two independent groups. Categorical variables were summarized as counts (%) and groups were compared using the Chi-square test or Fisher’s exact test as appropriate. Change over time across two groups was assessed using two-way analysis of variance (ANOVA) with repeated measures. Bonferroni adjustments were used to account for multiple analyses. Due to the lack of data regarding the relationship between changes in circumferential shortening and heart failure in this population, and the exploratory nature of this study, we used data from previous work with prospective case/control cohorts to estimate the sample size. Assuming an alpha of 0.05, 25 subjects per group would provide 80% power to detect 20% differences in RV longitudinal and circumferential shortening measures between groups equating to 4% change in absolute values. A *p* value less than 0.05 was used to determine significance. All results were considered statistically significant for values of *p* ≤ 0.05. A receiver operating characteristic curve was constructed to determine cut-off values for each measure with the best sensitivity and specificity to predict RV failure requiring OHT. The statistical analysis was performed using SPSS version 14.0 (SPSS, Inc., Chicago, IL, USA).

## 3. Results

### 3.1. Patients and Clinical Characteristics

From 783 records initially screened from our institutional single ventricle database, we identified 143 patients who underwent OHT. Of 143 patients, 26 had a diagnosis of HLHS, DORV, or UAVC and were followed up at *Time 3* and *4.* Of 26 study patients, 16 had consecutive data at *all* time points in our database. Of the 640 remaining patients in the database that did not receive OHT, we identified an equal number of matched controls at *all* time points that did not develop RV failure after the Fontan palliation. Both cohorts constituted 42% with HLHS, 27% with UAVC with dominant RV, and 31% with DORV. The median age and interquartile range at Fontan for the control group were similar to the study group (*p* = 0.06). There was no difference in cardiopulmonary bypass time (*p* = 0.62) between the study group and the control group. Demographic and clinical data are shown in Table 1.

The study group patients who eventually required OHT were initially at Stage A or Stage B heart failure like the control group at *Time 1,* but progressed to Stage C (fatigue, exercise intolerance, and fluid retention) after *Time 2.* Eleven study patients (42%) had mild RAVV regurgitation (annulus diameter mean 2.77 (0.45 cm). The remaining 58% of study patients had moderate insufficiency (annulus diameter mean 3.04 (0.63 cm), vena contracta width < 0.70 cm). There was no significant difference in RAVV annular dimension (*p* = 0.08) between the control and the study group throughout the time points. All of the study patients progressed to Stage C/D heart failure prior to developing complications of Fontan circulation. Eleven of the study patients (42%) exhibited symptoms of protein-losing enteropathy, of which four manifested arrhythmias. At *Time 3*, the study patients were on at least three medications including a β-blocker, ACE inhibitor, and diuretics and were listed for heart transplant with 1A or 1B status receiving continuous Milrinone infusion. Median age at OHT was 8.7 years (5.3–16.1) and the median interval from Fontan procedure to OHT was 6.6 years.

Control patients were categorized as either at risk for heart failure who have not yet developed structural heart changes (Stage A) or with evidence of asymptomatic mild ventricular dysfunction with no heart failure symptoms (Stage B) [18]. Thirteen (50%) of control patients had mild RAVV (annulus diameter mean 2.63 (0.46) cm). At *Time 3*, seven patients were on a single angiotensin-converting enzyme inhibitor (ACE) inhibitor and one patient was on a β-blocker and an ACE inhibitor. Fifteen patients were not on any medications.

### 3.2. RV Function

**Figure 3:**
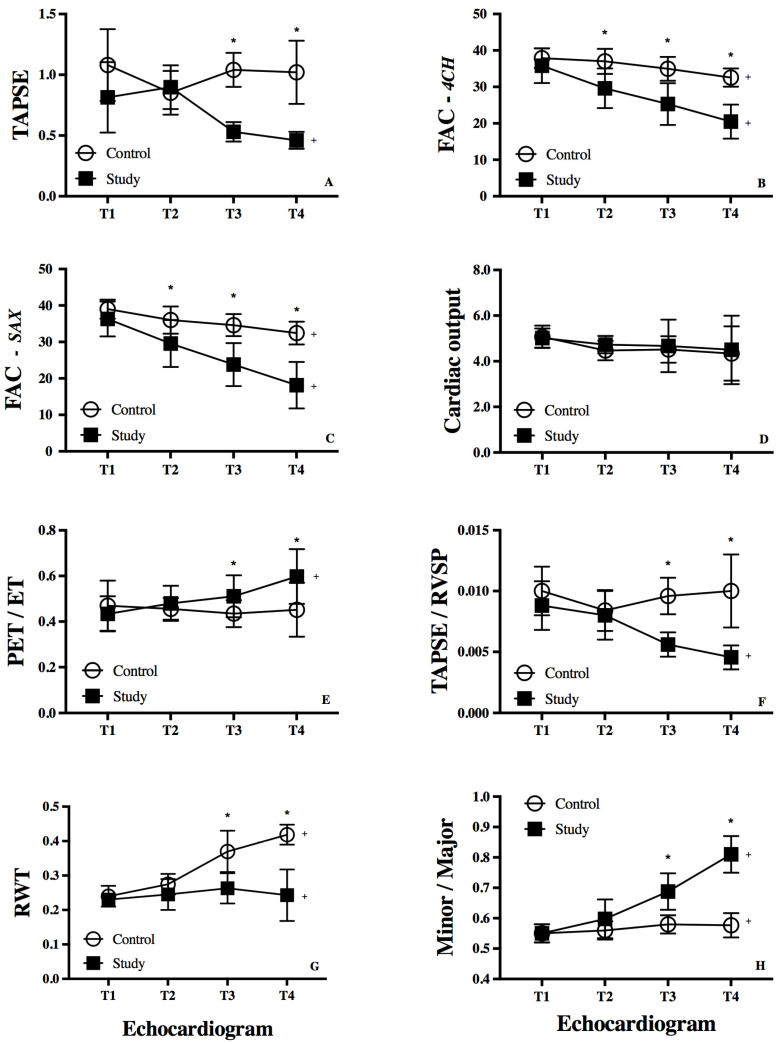
Comparison of functional and morphologic changes between controls and study patients. (**A**) Tricuspid annular plane systolic excursion (TAPSE, cm) between study (black boxes) and control (open circles); (**B**) Right ventricular fractional area of change (FAC, %); (**C**) RV FAC from the parasternal short-axis view (SAX); (**D**) Cardiac output (L/min); (**E**) Pre-ejection time/ejection time (PET/ET); (**F**) TAPSE/Right ventricular systolic pressure (RVSP) (cm/mmHg); (**G**) Relative wall thickness (RWT); (**H**) Minor/Major axis, quantifies the ratio between the RV minor-axis diameter parallel to the septum and the RV major-axis diameter perpendicular to the septum. * denotes *p* < 0.05 using *t* test comparing control vs. study group; + denotes *p* < 0.05 using univariate analysis of variance (ANOVA) between time points.

*Measures of RV longitudinal shortening*: TAPSE declined from *Time 1* to *4* in the study group (*p* < 0.01), and was also significantly lower at *Time 3* (*p* < 0.001) and *Time 4* (*p* = 0.01) compared to the control group (Table 2, Figure 3A). The trajectory of TAPSE was preserved in the control group (*p* = 0.14) from *Time 1* to *4*. RV longitudinal FAC from the four-chamber view declined in both groups from *Time 1* to *4,* but was significantly lower in the study group at *Time 2* (*p* < 0.001), *Time 3* (*p* < 0.001), and *Time 4* (*p* < 0.001) (Figure 3B). For the 16 patients in both groups with data for all time points, the findings of the analysis of TAPSE and RV longitudinal FAC were similar.

*Measures of RV circumferential shortening:* RV *circumferential* FAC from the parasternal short-axis view declined in both groups from *Time 1* to *4,* but was significantly lower in the study group at *Time 2* (*p* < 0.001), *Time 3* (*p* < 0.001), and *Time 4* (*p* < 0.001) (Table 2, Figure 3C). For the 16 patients in both groups with consecutive data for all time points, the findings of the analysis of RV circumferential were similar. There was no change in cardiac output from *Time 1* to *4,* and no difference between the cohorts at any stage (Figure 3D).

### 3.3. RV-Systemic Vascular Coupling

*Systolic time intervals:* The RV-PET and RVET were similar during *Time 1 and Time 2* between the groups (*p* > 0.05). However, the RV-PET was found to be statistically longer in the study than the control group at *Time 4* (*p* < 0.001). The RVET was significantly shorter in the study at *Time 4* (*p* < 0.001). The RV-PET/RVET ratio was higher in the study group at *Time 3* and *Time 4* (*p* < 0.001) (Table 2, Figure 3E). For the 16 patients in both groups with consecutive data for all time points, the findings of the analysis of RV-PET-RVET were similar.

*RV work:* RV-vascular coupling, denoted by the ratio TAPSE/RVSP, declined in the study group from *Time 1* to *4* (*p* < 0.01), and was lower at *Time 2* and *Time 3* (*p* < 0.01). (Table 2, Figure 3F). TASPE/RVSP remained stable from *Time 1* through *Time 4* in the control group (*p* = 0.17). For the 16 patients in both groups with consecutive data for all time points, the findings of the analysis of TASPE/RVSP were similar. 

### 3.4. RV Remodeling

*RV remodeling:* RWT increased from *Time 1 to 4* in the control group (*p* < 0.01), and was also significantly higher at *Time 3* (*p* < 0.001) and *Time 4* (*p* = 0.01) when compared to the study group. The trajectory of RWT was stagnant in the study group (*p* = 0.38) from *Time 1 to 4* (Table 2, Figure 3G). For the 16 patients in both groups with consecutive data for all time points, the findings of the analysis of RWT were similar.

*RV geometry:* The ratio of minor to major axis increased in the study group from *Time 1* to *4*, and was higher at *Time 3* (*p* < 0.001) and *Time 4* (*p* < 0.001). The minor/major axis was unchanged throughout the time points in the control group (Table 2, Figure 3G). For the 16 patients in both groups with consecutive data for all time points, the findings of the analysis of the minor/major were similar. Figure S5 is a parasternal short-axis view representing a control patient’s RV concentric remodeling compared to a more eccentric hypertrophy of a study patient’s RV.

### 3.5. Receiver Operating Characteristic (ROC) Curve Analysis

For detection of the need for OHT following Fontan, a longitudinal FAC < 30%, a circumferential FAC < 30%, TAPSE/RVSP < 0.05, a minor/major-axis ratio > 0.6, and a RWT < 0.3 resulted in combined sensitivity of 87% and specificity of 88% with an area under the curve of 0.91 (0.83–0.97). The best cut-off value for predicting OHT was a minor/major > 0.6 (AUC 0.94, 95% CI 0.85–0.97) followed by an RWT < 0.3 (AUC 0.91, 95% CI 0.83–0.95) (Figure S6).

### 3.6. Reproducibility

We demonstrated excellent inter- and intra-observer reliability with narrow limits of agreement, a relative bias < 10% and a coefficient of variation < 10% for all measures. In addition, the reproducibility was similar between the DORV and HLHS groups at both time points.

## 4. Discussion

The main findings in this longitudinal study to characterize the trajectory of systemic RV performance after the Fontan procedure are that RV failure developed from progressive decline in RV systolic function in patients who failed to develop positive RV remodeling to loading conditions. These results highlight the importance of interpreting RV function in the context of its load. Enhanced RV circumferential shortening preserved RV geometry, and RV-vascular coupling all reflect structural and functional remodeling as protective mechanisms against RV heart failure after the Fontan procedure. These results add to the emerging list of clinically relevant noninvasive quantifiable markers of disease evolution of heart failure in Fontan patients.

### 4.1. RV Remodeling and RV-Systemic Vascular Coupling

Although morphological RV as a systemic ventricle in the Fontan patients is a risk factor for long-term survival [1,2,3], there have been few mechanistic studies investigating RV performance leading to failure in the context of Fontan hemodynamics. Our study presents novel findings of a continuum of different patterns of RV remodeling in Fontan patients who developed progressive RV dysfunction and failure requiring heart transplantation. RV morphometric measurements demonstrated a more concentric remodeling with significantly increasing RWT (a surrogate for higher mass-to-volume ratio) > 0.3 in control group, whereas a more eccentric hypertrophy with lower RWT (a surrogate for lower mass-to-volume ratio) < 0.3 in the study group of Fontan patients by *Time 3* on the time trajectory (Figure 3G). Measures of absolute RV wall thickness and wall thickness relative to RV volume have been shown to increase after the hemi-Fontan procedure in HLHS infants denoting a marked change in RV geometry [19]. The globular geometry with increasing minor/major-axis ratio in the study group likely induced greater RV wall stress (Laplace’s law) for a given pressure increase comparable to Fontan patients in the control group. Because of the smaller RWT of the SRV in the study group, wall stress (the afterload) was not commensurately compensated resulting in ventricular contractility-afterload mismatch, similar to when contractility of the hypertrophied RV is insufficient to compensate for the increase in RV afterload in pulmonary hypertension [20]. This is manifested by RV-vascular uncoupling with a decreasing TAPSE/RVSP ratio. The RV-PET/RVET was also progressively prolonged in the study group, indicating a longer portion of the cardiac cycle in isovolumetric contraction, and a shorter portion in ejection due to increased RV contractility-vascular load mismatch [21]. Similar to patients with severe pulmonary hypertension characterized by systemic RV pressure and RV failure (a pathophysiologic condition affecting the RV similar to those in our cohort), the RV-PET/RVET was higher prior to intervention and decreased after Potts shunt creation (anastomosis between the descending aorta and left pulmonary artery) because of a decrease in afterload [21].

TAPSE/RVSP, as an index of RV-vascular coupling, provides a noninvasive assessment of RV work and its contractile state [12,22]. In this study, the TAPSE/RVSP ratio progressively declined in the Fontan patients of the study group, but remained relatively stable after *Time 2* in the control group. Most of the Fontan patients in the study group had a TAPSE/RVSP ratio ≤ 0.05 compared to the ≥0.08 in the Fontan patients in the control group and developed Stage C heart failure by (AHA heart failure classification) [18]. A contractility-afterload mismatch has been attributed to increased impedance in Fontan circulation secondary to the additional connection of the pulmonary vascular bed to the systemic vasculature raising vascular resistance (the nonpulsatile load on the ventricle), low-frequency impedance (pulsatile load on the ventricle), and deterioration in myocardial contractility [23,24]. However, in studies of Fontan patients with both RV and LV morphology, depressed contractility was not inherent in Fontan physiology and was associated with limited inotropic response and decreased beta-adrenergic reserve owing to limited preload [25]. Our study reveals that early adaptive remodeling in Fontan patients of the control group allowed for preservation of SRV systolic function and TAPSE/RVSP coupling despite chronic hemodynamic overload.

### 4.2. Systemic RV Systolic Function in Fontan Patients

In the control group, the systemic RV remodeled with higher circumferential systolic function, mimicking the LV contraction pattern. The geometric changes in the SRV with increased circumferential shortening and radial thickening are typical of how the LV remodels by concentric hypertrophy and compensates for chronic pressure overload with biventricular physiology [26]. This suggests that the remodeling of the SRV may be due to the development of dominance (hypertrophy) of myofiber architecture composed of the transverse layers of the free wall [27]. The RV function in SRV due to HLHS depends on the free wall’s intrinsic contraction capacity, its contracting pattern relative to the septum, and the capacity of the septum to be a buttress with depressed free wall function [28]. Since the septum contains right-angle cross-striation of longitudinal fibers, the circumferential pattern of contraction that takes place as the compensatory mode of deformation is consistent with the predominance of transverse muscle fiber that surrounds the RV free wall. The enhanced circumferential shortening seen in the Fontan patients of the control group reflects the important adaptive process when facing chronic systemic overload. We infer from our study that the inability of the SRV in the study group to remodel its transverse myoarchitecture and adapt its contraction patterns was reflective of maladaptive remodeling contributing to heart failure due to the trajectory of declining SRV systolic function after the Fontan procedure. Finally, we suspect that cardiac output was maintained despite a decrease in RF FAC due to preserved stroke volume with compensatory mechanisms, remodeling with RV dilation, and/or changes in loading conditions rather than intrinsic contractile decline. More investigation is needed to delineate this observation.

This is the first study to describe circumferential shortening depicted by RV FAC from the parasternal short-axis view. Other reports have shown similar findings of increased circumferential shortening with strain pattern changes in RV function in patients with congenital heart disease. Khoo et al. reported findings of increased magnitudes of circumferential strain compared to longitudinal strain in HLHS between the first two stages of surgical palliation [29]. Further, Lin et al. showed that children with HLHS with normal four-chamber RV FAC and strain parameters before bidirectional Glenn have a low likelihood of death or heart transplant over the next 5 years [30].

Our study does not provide an explanation why the SRV failed to remodel, or at some point remodeling became maladaptive in the study group leading to a decline in SRV function and progression to RV failure. Significant RAVV regurgitation adding volume overload to SRV, is a causative factor for maladaptive remodeling [31]. In the study group, there were more patients with more than mild RAVV regurgitation. However, we found that there was no difference in the RAVV annulus throughout time points in each patient of both groups reflecting that volume overload was not causing RAVV dilatation and regurgitation. The AV valve regurgitation may primarily be a consequence of SRV free wall, septal dyssynchrony [32], or planar tricuspid annulus rather than from annular dilatation of the AV valve from volume overload alone [33]. Taken together with our findings, enhanced circumferential shortening may be a valuable marker of adaptive response or myocardial dysfunction in the systemic ventricle. Identifying the earliest transition point from adaptive to maladaptive may be the “Holy Grail” of patient assessment.

### 4.3. Study Limitations

This study has important limitations. Causality cannot be proven from the retrospective, nonrandomized design, and the results should be interpreted within the framework of these study limitations. However, the study does provide a proven hypothesis that can serve as the basis for a larger prospective study. Although the study was properly powered, it included a small number of patients in each group, limiting our ability to account for all the different confounders related to the disease and the echocardiographic measures. This study is based on physiologic interpretation of echocardiographic parameters and did not incorporate biomarkers of heart failure given that this was a retrospective study. However, studies have shown that biomarkers such as brain natriuretic peptide have limited utility in the Fontan population [34]. This study did not assess other noninvasive surrogates of RV contractility, such as myocardial strain parameters. These indexes may provide incremental diagnostic and prognostic information in infants [35] and adults [36] with congenital heart diseases. Further prospective studies evaluating the myocardial deformation properties of the systemic right ventricle in Fontan patients are needed. Finally, we hope that this work can be integrated into future predictive models that utilize multimodality imaging in patients with systemic right ventricles [37,38].

## 5. Conclusions

In conclusion, our study infers that systemic RV remodeling with enhanced RV circumferential systolic function and preserved RV-vascular coupling appears to be a positive adaptive and protective mechanism against RV heart failure in Fontan patients. Longitudinal monitoring of RV circumferential systolic shortening along with indices of cardiac remodeling and RV-vascular coupling may serve as clinically relevant quantifiable markers of disease evolution, and early indicators for the need for interventions for progressive RV failure after the Fontan procedure for the univentricular RV physiology.

## Figures and Tables

**Table 1:** Comparison of Demographic and Clinical data between Control and Study groups.

Timing for Echocardiogram	Time 1	Time 2	Time 3	Time 4
Control Patients (*n* = 26)				
Age at Echo (Years)	3.7 (3.1–7.5)	3.8 (3.2–6.7)	6.2 (5.2–7.9)	7.8 (4.6–14.8)
Weight (kilograms)	15.8 (14.8–37.7) *	16.2 (15.3–20.1)	18.1 (16.0–27.4)	26.5 (17.8–48.6)
Body Surface Area (m^2^)	0.82 (0.64–1.0) *	0.68 (0.63–0.83)	0.9 (0.7–1.0)	1.0 (0.7–1.5)
Heart Rate (bpm)	93 (74–109)	95 (77–103)	77 (66–98)	76 (71–94) *
SBP (mmHg)	96 (86–104)	92 (91–98)	104 (100–110)	99 (90–114)
DBP (mmHg)	54 (54–65)	52 (50–57)	52 (51–63)	60 (54–69)
Study Patients (*n* = 26)				
Age at Echo (Years)	3.2 (3.0–4.2)	4.0 (3.7–7.4)	7.8 (6.5–12.5)	9.0 (5.6–16.4)
Weight (grams)	18.1 (15.6–20.5)	19 (18–26)	22.3 (19.0–34.0)	28.4 (18.0–46.8)
Body Surface Area (m^2^)	1.4 (1.1–1.4)	0.9 (0.7–1.0)	0.9 (0.7–1.1)	1.0 (0.7–1.4)
Heart Rate (bpm)	79 (78–85)	90 (85–116)	105 (87–118)	98 (85–118)
SBP (mmHg)	87 (87–165)	100 (93–115)	95 (91–105)	99 (86–108)
DBP (mmHg)	53 (53–67)	56 (53–58)	56 (54–59)	59 (51–67)

Data expressed as median (Interquartile range, IQR). * *p* < 0.01 Control vs. Study patients.

**Table 2:** Comparison of Echocardiography Variables between the Control and Study Groups.

Timing for Echocardiogram	Time 1	Time 2	Time 3	Time 4	*p*-Value
Control Patients (*n* = 26)					
RV performance					
FAC 4CH (%)	38 (36–39)	36 (35–39) *	34 (32–38) *	31 (30–33) *	0.01
FAC SAX (%)	39 (38–40)	35 (34–38) *	35 (33–36) *	30 (30–34) *	<0.01
TAPSE (cm)	1.1 (0.8–1.2)	0.8 (0.7–1.1)	1.0 (0.9–1.1) *	0.9 (0.8–1.0) *	0.09
TAPSE/RVSP	0.1 (0.08–0.12)	0.08 (0.07–0.09)	0.08 (0.08–0.10) *	0.08 (0.08–0.1) *	0.17
RV-PET/ET	0.43 (0.40–0.49)	0.43 (0.42–0.49)	0.45 (0.41–0.48) *	0.46 (0.40–0.50) *	0.10
Hemodynamics					
CO (L/min)	4.9 (4.8–5.5)	4.5 (4.2–4.5)	4.3 (4.1–4.8)	4.2 (3.3–5.1)	0.22
SV (ml/m^2^)	69 (65–81)	55 (51–67) *	54.4 (48.0–65.8)	53.0 (39.7–68.4)	0.04
RV geometry					
RWT (cm)	0.25 (0.23–026)	0.27 (0.25–0.27)	0.31 (0.29–0.34) *	0.42 (0.41–0.43) *	<0.01
Minor/Major Axis	0.57 (0.51–0.58)	0.57 (0.55–0.59)	0.57 (0.56–0.59) *	0.57 (0.55–0.62) *	0.09
Study Patients (*n* = 26)					
RV performance					
FAC 4CH (%)	34 (32–40)	28 (26–32)	28 (21–30)	21 (18–24)	<0.01
FAC SAX (%)	38 (35–38)	30 (24–33)	21 (19–30)	19 (12–22)	<0.01
TAPSE (cm)	0.9 (0.8–1)	0.7 (0.6–1.1)	0.6 (0.5–0.6)	0.45 (0.4–0.5)	<0.01
TAPSE/RVSP	0.08(0.07–0.08)	0.08 (0.05–0.10)	0.05 (0.05–0.0	0.04 (0.04–0.05)	<0.01
RV-PET/ET	0.44 (0.38–0.49)	0.47 (0.43–0.51)	0.51 (0.45–0.57)	0. 60 (0.53–0.67)	<0.01
Hemodynamics					
CO (L/min)	4.8 (4.6–5.5)	4.4 (4.4–5.1)	4.5 (4.1–5.3)	4.4 (3.5–5.4)	0.66
SV (ml/m^2^)	72 (69–78)	45 (42–58)	48.2 (42.6–59.5)	42.3 (34.8–53.7)	<0.01
RV geometry					
RWT (cm)	0.24 (0.22–0.24)	0.24 (0.21–0.27)	0.26 (0.22–0.30)	0.24 (0.20–0.28)	0.62
Minor/Major Axis	0.56 (0.53–0.58)	0.59 (0.58–0.63)	0.68 (0.64–0.71)	0.80 (0.77–0.85)	<0.01

Data expressed as median (Interquartile range, IQR). Fractional area of change (FAC); Four-chamber view (4CH); Short-axis view (SAX); Tricuspid annular plane excursion (TAPSE); RV systolic pressure (RVSP); Pre-ejection time (PET); Ejection time (ET); CO, cardiac output; SV, stroke volume. RWT, relative wall thickness. * *p* < 0.01 Control vs. Study patients: *p*-value is based on repeated measures ANOVA with Bonferroni adjustment.

## Data Availability

Data is unavailable due to privacy.

## Supplementary Materials

The following supporting information can be downloaded at https://www.mdpi.com/article/10.3390/jcm14134602/s1, Figure S1: Tricuspid annular plane systolic excursion; Figure S2: Right ventricular systolic time intervals; Figure S3: Relative wall thickness; Figure S4: Minor and major axes; Figure S5: Right ventricle remodeling; Figure S6: ROC curve for orthotopic heart transplant (OHT).

## Abbreviations

The following abbreviations are used in this manuscript:

RV	Right ventricular
LV	Left ventricle
SRV	Single right ventricular morphology
TAPSE	Tricuspid annular plane systolic excursion
OHT	Orthotopic heart transplant
DORV	Double outlet right ventricle
UAVC	Unbalanced atrioventricular canal
HLHS	Hypoplastic left heart syndrome
FAC	Fractional area of change
RVESA	RV end-systolic area
RVEDS	RV end-diastolic area
AV	Atrioventricular valve
RVET	RV ejection time
RV-PET	RV pre-ejection time
RVSP	RV systolic pressure
SBP	Systolic blood pressure
DBP	Diastolic blood pressure
RWT	Relative wall thickness
RVID	RV internal diameter
ILWT	Inferolateral wall thickness
ACE	Angiotensin-converting enzyme inhibitor
ROC	Receiver operating characteristic
RVEDS	RV end-diastolic area
