# Supplementary material for: Evolution and Predictors of Right Ventricular Failure in Fontan Patients: A Case-Control Study
Source: J Clin Med. 2025 Jun 29;14(13):4602. doi: 10.3390/jcm14134602 (PMC12250303; doi:10.3390/jcm14134602)
Supplement: Supplementary file 1 [file jcm-14-04602-s001.zip › jcm-3700533-supplementary.pdf]

Journal of
Clinical Medicine

MDPI

Supplemental Material

Evolution and Predictors of Right Ventricular Failure in Fontan Patients: A Case-Control Study

Hannah S. Kim [1], Ginnie Abarbanell [2], Kathleen Simpson [3], Aaron M Abarbanell [4], Pirooz Eghtesady [5], Philip T Levy [6,*], Gautam K. Singh [7]

1 Department of Pediatrics, Hackensack Meridian School of Medicine, Nutley, New Jersey. hannah.kim@hmhn.org
2 Department of Pediatrics, UT Health, San Antonio ; abarbanell@uthscsa.edu
3 Department of Pediatrics, University of Colorado Denver, Children's Hospital of Colorado, Denver, Colorado, Kathleen.Simpson@Childrenscolorado.org
4 Department of Cardiothoracic Surgery, UT Health, San Antonio. abarbanella@uthscsa.edu
5 St. Louis Children's Hospital, Pediatric Cardiothoracic Surgery; eghtesady670@wustl.edu
6* Division of Newborn Medicine, Boston Children's Hospital, Department of Pediatrics, Harvard Medical School, Boston, Massachusetts. Philip.levy@childrens.harvard.edu
7 Division of Pediatric Cardiology, Children's Hospital Michigan and Central Michigan University, Detroit, Michigan; GSingh3@dmc.org

Supplemental Figures

Figure S1: Tricuspid annular plane systolic excursion ……………………..……..Page 2

Figure S2: Right ventricular systolic time intervals……………………………..….Page 2

Figure S3: Relative wall thickness…………………………………………………....Page 2

Figure S4: Minor and major axes. …………………………………………………....Page 3

Figure S5: Right ventricle remodelling………………………………………………Page 3

Figure S6: ROC curve for orthotopic heart transplant (OHT)……………………..Page 4

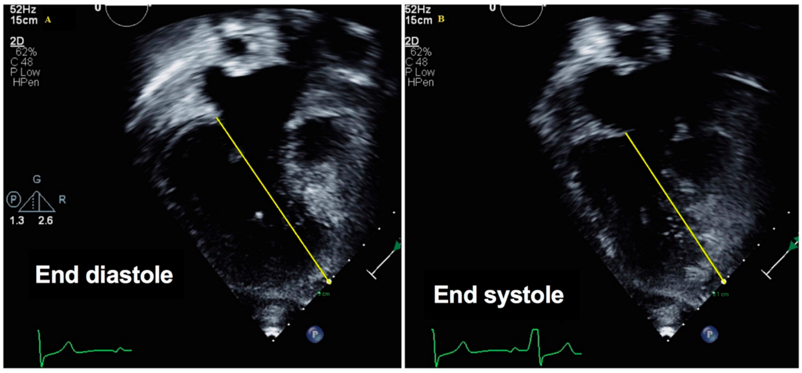

Figure S1. Tricuspid annular plane systolic excursion. Tricuspid annular plane systolic excursion (TAPSE) was measured by calculating the change in distance from the lateral annulus of the right AV valve to a constant fixed apical point at end-diastole to end-systole.

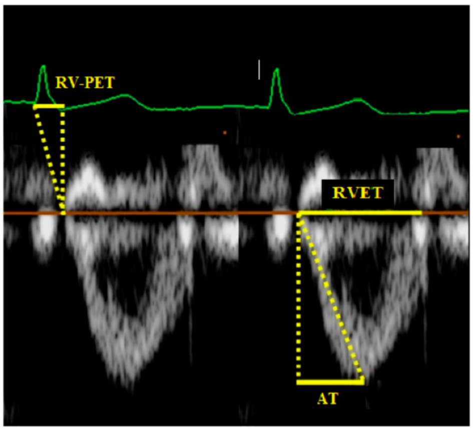

Figure S2. Right ventricular systolic time intervals. RV pre-ejection time (RV-PET) was measured from the Q wave of the electrocardiogram tracing to the beginning of the neo-aortic Doppler waveform. RV ejection time (RVET) was measured form the beginning to the termination of the neo-aortic flow spectrum.

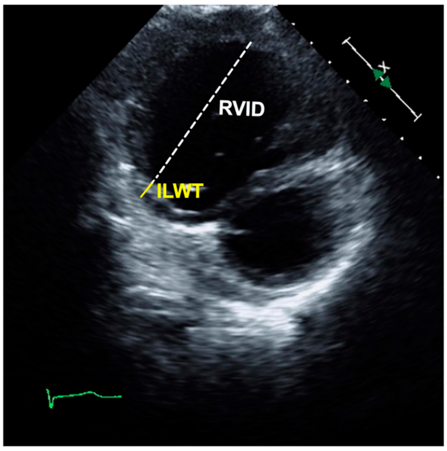

Figure S3. Relative wall thickness. Relative wall thickness (RWT) was calculated using the ratio of twice the RV inferolateral wall thickness (ILWT) constituted by the compacted

myocardium, to the RV internal diameter (RVID) measured at the end-diastole at the level of the anterior and septal papillary muscles.

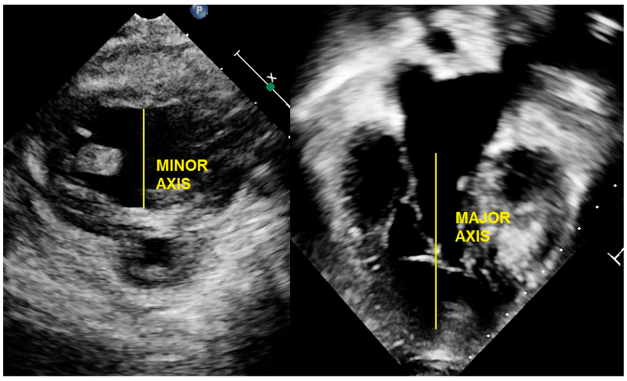

Figure S4. Minor and Major Axes. RV geometry was determined by using the ratio of minor to major axes. Major axis was measured from the midpoint of the right AVV annulus to the apex in the apical four-chamber view. Minor axis was the mid RV internal dimension at the level of the anterior and septal papillary muscles in the short axis view at end-diastole.

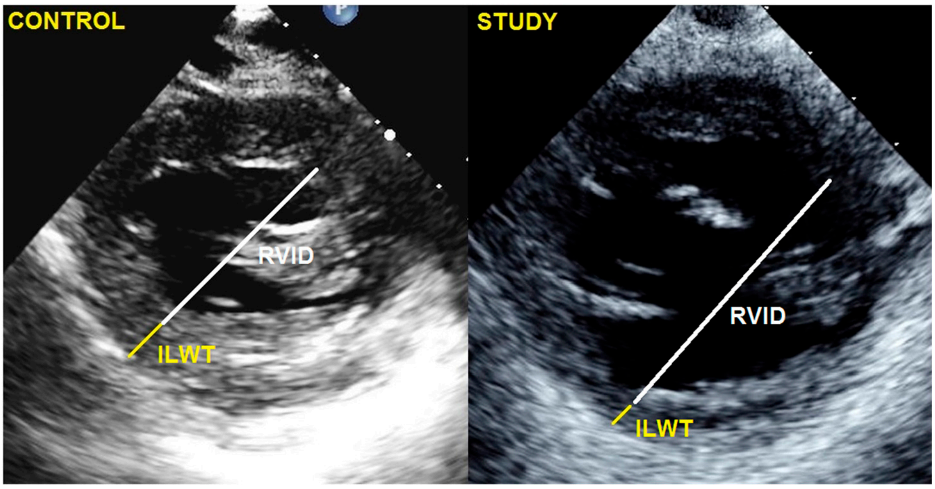

Figure S5. RV remodeling. Parasternal short-axis view representing a control patient's RV concentric remodeling (Left) compared to a more eccentric hypertrophy of a study patient's RV (Right). Infero-lateral wall thickness (ILWT); Right ventricular internal diameter (RVID).

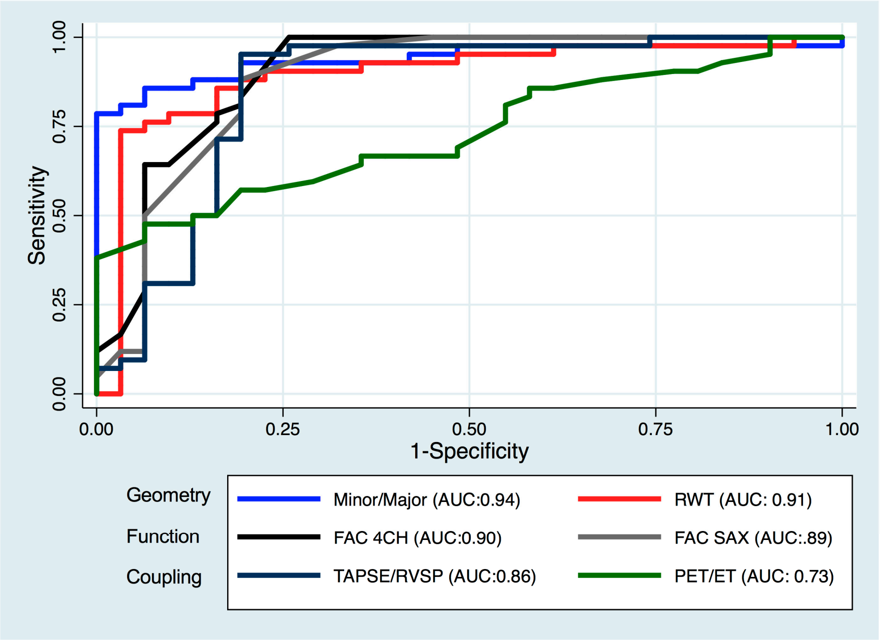

Figure S6. Receiver operating characteristic (ROC) curve for orthotopic heart transplant (OHT). For detection of the need for OHT following Fontan, a longitudinal FAC <30%, a circumferential FAC <30%, TAPSE/RVSP <0.05, a minor/major axis ratio > 0.6, and a RWT <0.3 resulted in combined sensitivity of 87% and specificity of 88% with an area under the curve of 0.91 (0.83-0.97).
